# Fludarabine treatment favors the retention of miR-485-3p by prostate cancer cells: implications for survival

**DOI:** 10.1186/1476-4598-12-52

**Published:** 2013-06-05

**Authors:** Serena Lucotti, Giuseppe Rainaldi, Monica Evangelista, Milena Rizzo

**Affiliations:** 1 Laboratory of Gene and Molecular Therapy, Institute of Clinical Physiology, Area della Ricerca CNR,Via Moruzzi,1, Pisa 56124, Italy; 2 Istituto Toscano Tumori, Firenze, Italy

**Keywords:** Circulating miRNAs, DU-145 prostate cancer cell line, Drug sensitivity, Exosomes

## Abstract

**Background:**

Circulating microRNAs (miRNAs) have been found in many body fluids and represent reliable markers of several physio-pathological disorders, including cancer. In some cases, circulating miRNAs have been evaluated as markers of the efficacy of anticancer treatment but it is not yet clear if miRNAs are actively released by tumor cells or derive from dead tumor cells.

**Results:**

We showed that a set of prostate cancer secretory miRNAs (PCS-miRNAs) were spontaneously released in the growth medium by DU-145 prostate cancer cells and that the release was greater after treatment with the cytotoxic drug fludarabine. We also found that the miRNAs were associated with exosomes, implying an active mechanism of miRNA release. It should be noted that in fludarabine treated cells the release of miR-485-3p, as well as its association with exosomes, was reduced suggesting that miR-485-3p was retained by surviving cells. Monitoring the intracellular level of miR-485-3p in these cells, we found that miR-485-3p was stably up regulated for several days after treatment. As a possible mechanism we suggest that fludarabine selected cells that harbor high levels of miR-485-3p, which in turn regulates the transcriptional repressor nuclear factor-Y triggering the transcription of topoisomerase IIα, multidrug resistance gene 1 and cyclin B2 pro-survival genes.

**Conclusions:**

Cytotoxic treatment of DU-145 cells enhanced the release of PCS-miRNAs with the exception of miR-485-3p which was retained by surviving cells. We speculate that the retention of miR-485-3p was a side effect of fludarabine treatment in that the high intracellular level of miR-485-3p plays a role in the sensitivity to fludarabine.

## Background

MicroRNAs (miRNAs) are released in the body fluids and due to their abundance and stability they can be easily detected and/or monitored over long periods [1-5]. If either physiological or pathological disorders affect the human body, the miRNA signature of body fluids is shifted, making them useful diagnostic/prognostic markers for a given disorder [6-11]. Prostate cancer patients have been found to have specific miRNAs (prostate cancer secretory miRNAs, PCS-miRNAs) overrepresented in their plasma/serum, these are currently considered potential markers for the disease [3,12-17]. In some cases, circulating miRNAs have been evaluated as markers of the efficacy of anticancer treatment [17], but it is not yet clear if miRNAs are actively released by tumor cells or derive from dead tumor cells. We approached this aspect using cancer cell lines, as they offer the possibility to determine both intracellular and extracellular miRNAs, thus allowing investigation of whether cancer cells treated with a cytotoxic drug release miRNAs in the growth medium like untreated cells.

Here we report that DU-145 prostate cancer cells release a set of PCS-miRNAs and that after a treatment with fludarabine, a drug which preferentially blocks the progression of cells toward S phase and later induces cell death [18], the release of PCS-miRNAs was enhanced with the exception of miR-485-3p which was retained by surviving cells. We speculate that the reduced release of miR-485-3p was a side effect of fludarabine treatment and discuss the potential role of this miRNA in determining the sensitivity of DU-145 cells to the drug.

## Results

### PCS-miRNAs are released into the growth medium by the DU-145 tumor cells

**Figure 1:**
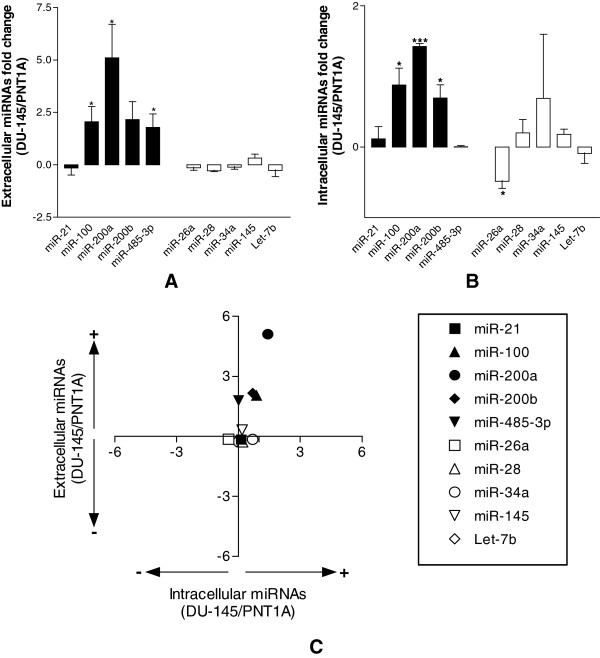
**PCS- and S-miRNAs levels in the growth medium of DU-145 cells.** Extracellular (**A**) and intracellular (**B**) expression levels of PCS-miRNAs (black bars) and S-miRNAs (white bars) in DU-145 cells in comparison to PNT1-A cells. The fold change of each miRNA was calculated by subtracting the expression value of PNT1-A cells from that of DU-145 cells and then normalizing with the value of PNT1-A cells. **C**, dot plot of intracellular (Intra) to extracellular (Extra) levels of PCS- and S-miRNAs, expressed as fold changes in DU-145 in comparison to PNT1A cells. *Upper left quarter*: Intra down regulated, Extra up regulated; *Upper right quarter*: Intra up regulated, Extra up regulated; *Lower left quarter*: Intra down regulated, Extra down regulated; *Lower right quarter*: Intra up regulated, Extra down regulated. All data were shown as S.D. from three independent experiments (*P<0.05, **P<0.01, ***P<0.001, unpaired *t*-test).

To build a reliable *in vitro* model to study miRNA release, we verified whether the DU-145 prostate cancer cell line spontaneously releases miRNAs into the growth medium. To address this question we considered 5 prostate cancer secretory miRNAs (PCS-miRNAs) and 5 secretory miRNAs (S-miRNAs) representative respectively of the miRNAs overrepresented or not overrepresented in the plasma/serum of patients with prostate cancer [3,14,16]. We found that in comparison to the immortalized prostate epithelial cell line PNT1-A, all PCS-miRNAs (with the exceptions of miR-21) were more abundant in the growth medium of DU-145 cells, unlike S-miRNAs (Figure 1A). These data indicate that DU-145 cells were able to release the same miRNAs that are overrepresented in the plasma of prostate tumor patients.

We then asked if the release of miRNAs was influenced by their endogenous expression levels. We quantified the intracellular levels of our panel of miRNAs and found elevated intracellular levels of almost all PCS-miRNAs in comparison to PNT1A cells (Figure 1B). The dot plot of the intracellular versus the extracellular levels showed a positive correlation for many PCS-miRNAs whereas this correlation was not observed for S-miRNAs (Figure 1C) suggesting a positive relationship between the intracellular and the extracellular levels for PCS-miRNAs.

### The release of PCS-miRNAs is enhanced by a cytotoxic treatment

**Figure 2:**
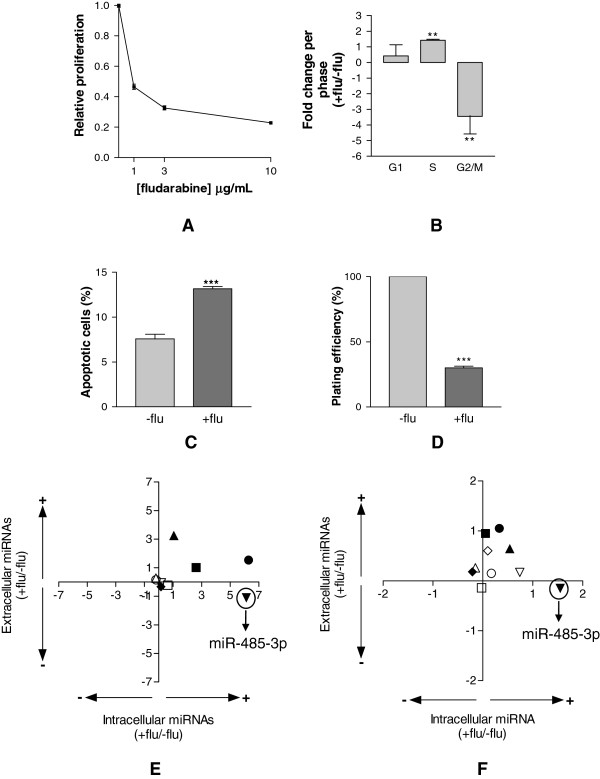
**Effects of fludarabine on DU-145 cells. A**, relative cell proliferation of DU-145 cells exposed for 48 h to increasing concentrations (0-10 μg/ml) of fludarabine. Cell cycle analysis (**B**), apoptosis (**C**) and plating efficiency (**D**) of DU-145 cells exposed for 48 h to 10 μg/ml of fludarabine. Dot plot of intracellular (Intra) to extracellular (Extra) levels of PCS- and S-miRNAs, expressed as fold changes in treated in comparison to untreated DU-145 cells with 10 μg/ml (**E**) or 1 μg/ml (**F**) of fludarabine. *Upper left quarter*: Intra down regulated, Extra up regulated; *Upper right quarter*: Intra up regulated, Extra up regulated; *Lower left quarter*: Intra down regulated, Extra down regulated; *Lower right quarter*: Intra up regulated, Extra down regulated. All data were shown as S.D. from three independent experiments (*P<0.05, **P<0.01, ***P<0.001, unpaired *t*-test).

We investigated the release of miRNAs after the exposure of DU-145 cells to a cytotoxic drug. We selected fludarabine, a drug which we have already used to induce cytotoxicity in tumor cells, as representative of cytotoxic drugs [19]. DU-145 cells were exposed to increasing concentrations of fludarabine for 48 h and from the dose-response curve we selected 10 μg/ml fludarabine as at this concentration cell proliferation was inhibited (Figure 2A). The cell-cycle analysis of DU-145 treated cells showed a blockage of cells in S and a strong depletion of G2 cells (Figure 2B). A slight but significant increase of apoptotic cells was also found (Figure 2C). The finding that after fludarabine treatment a substantial fraction (25%) of DU-145 cells was able to form colonies (Figure 2D) suggested that there were many surviving cells. Therefore, at the end of 48 h treatment we collected both attached cells and growth medium and measured the levels of our sets of miRNAs and calculated fold change of expression in treated versus untreated samples. The dot plot of the intracellular versus the extracellular fold changes showed that, while S-miRNAs had little variations after fludarabine treatment, almost all PCS-miRNAs were up regulated and released into the growth medium with the exception of miR-485-3p, which was less released by tumor cells despite its intracellular up regulation (Figure 2E). Interestingly, decreasing the concentration of fludarabine to 1 μg/ml, a concentration which reduced cell proliferation by 50% (Figure 2A), miR-485-3p was still retained (Figure 2F).

In order to define if miR-485-3p retention was common in prostate cancer cell lines, we extended the analysis of retention/release of miR-485-3p to PC-3, a prostate cancer androgen resistant cell line, and 22Rv1 and LNCaP, two androgen sensitive prostate cancer cell lines. Following the treatment with a concentration of fludarabine able to inhibit cell proliferation, we found that miR-485 was released in the growth medium of PC-3, 22Rv1 and LNCaP (Additional file 1: Figure S1A, B, C respectively), suggesting that the induction of release/retention is a process dependent on the genetic background. Then we asked whether the process was drug specific. We treated DU-145 cells with taxotere, a drug used for the treatment of prostate cancer. The dot plot of intracellular versus extracellular fold change of miRNAs levels showed that taxotere did not affected miR-485-3p retention/release (Additional file 1: Figure S1D) introducing the concept that a signature interaction between drug and genetic background exists.

### Exosomes are involved in the release of PCS-miRNAs

**Figure 3:**
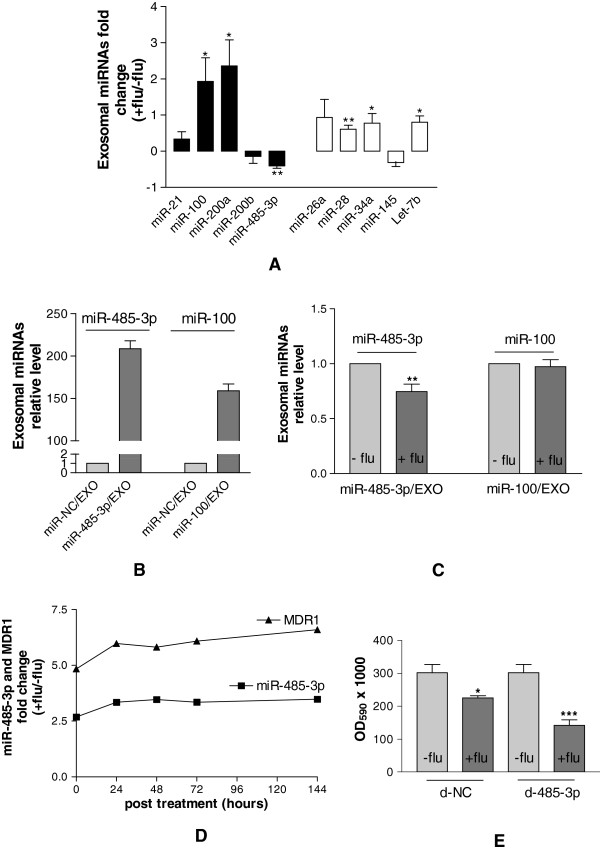
**PCS- and S-miRNAs levels in the exosomes isolated from DU-145 cells growth medium*. *A**, fold change of PCS- (black bars) and S- (white bars) miRNAs levels in exosomes isolated from the growth medium of DU-145 fludarabine treated in comparison to untreated cells. **B**, relative miR-485-3p or miR-100 levels in exosomes of DU-145 cells transfected with miR-485-3p and miR-100 respectively versus those of miR-NC transfected cells. **C**, relative miR-485-3p or miR-100 levels in exosomes of DU-145 cells transfected with miR-485-3p or miR-100 respectively and then treated with fludarabine versus those of transfected but untreated cells. **D**, fold change of the expression levels of MDR1 and miR-485-3p in DU-145 cells at different time points after fludarabine treatment. **E**, cell proliferation of DU-145 cells transfected with antagomiRNA-485-3p (d-485-3p) or antagomiRNA-NC (d-NC) and after having been exposed (+flu) or unexposed (-flu) to fludarabine for 48 h. All data were shown as S.D. from three independent experiments (*P<0.05, **P<0.01, ***P<0.001, unpaired *t*-test).

To address if the release of PCS-miRNAs enhanced by fludarabine occurs through an active mechanism, we detected their loading on exosomes. We isolated exosomes from the growth medium of DU-145 cells treated (EXO-flu^+^) or untreated (EXO-flu^-^) with fludarabine. Once we had verified that the amounts of EXO-flu^+^ and EXO-flu^-^ were similar and that exosomes were functionally active (Additional file 2: Figure S2), we determined the fold change of PCS-miRNAs and S-miRNAs in EXO-flu^+^ compared to EXO-flu^-^. We found that fludarabine favored the association of almost all PCS-miRNAs and S-miRNAs to EXO-flu^+^ compared to EXO-flu^-^ with the exception of miR-485-3p (Figure 3A). MiR-485-3p was released less by treated cells and less loaded on EXO-flu^+^ than on EXO-flu^-^ suggesting that fludarabine affects the association of miR-485-3p with exosomes.

To support this hypothesis, DU-145 cells were transfected with either miR-485-3p or miR-100 and the expression of miRNAs was measured in the exosomes released by transfected cells treated (miR-485-3p/EXO-flu^+^ or miR-100/EXO-flu^+^) or untreated (miR-485-3p/EXO-flu^-^ or miR-100/EXO-flu^-^) with fludarabine. Firstly, we observed that the two miRNAs were highly loaded on exosomes isolated from transfected cells (Figure 3B). When transfected cells were treated with fludarabine, we found that miR-485-3p/EXO-flu^+^ were less loaded with miR-485-3p than miR-485-3p/EXO-flu^-^ whereas miR-100/EXO-flu^+^ were loaded with miR-100 to a similar extent to miR-100/EXO-flu^-^ (Figure 3C). Since the association of miR-485-3p with exosomes was efficient in untreated cells but inhibited in fludarabine surviving cells, the possible explanations are that fludarabine alter the availability of miR-485-3p for the export or, alternatively, that fludarabine selected cells with a reduced ability to associate miR-485-3p with exosomes. In both cases the final result is that surviving cells were those enriched for miR-485-3p.

To investigate whether cells harboring high levels of miR-485-3p were induced or selected by fludarabine, we monitored the expression of miR-485-3p and its indirect target multidrug resistance gene 1 (MDR1) for 144 h (6 days) in fludarabine surviving cells. At the beginning of the treatment (t=0) the expression levels of miR-485-3p and MDR1 were respectively 2.5 and 5 fold higher in treated than in untreated cells and the high expression levels were stably maintained to late time points (Figure 3D). Due to the stable up regulation, it seems that fludarabine selected cells with a high intracellular level of miR-485-3p. To clarify this apparent relationship DU-145 cells were first transfected with an antagomiRNA specific for miR-485-3p and then treated with fludarabine. We found that cells depleted of miR-485-3p were more sensitive to fludarabine (Figure 3E) thus establishing that miR-485-3p was a modulator of the sensitivity to fludarabine.

### miR-485-3p protects DU-145 cells from the antiproliferative effects of fludarabine

**Figure 4:**
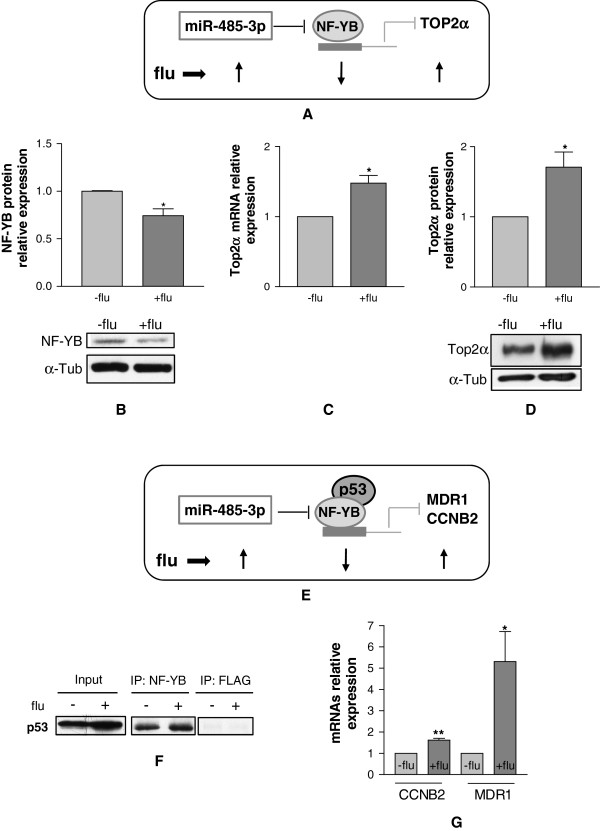
**Molecular characterization of fludarabine surviving cells. A**, schematic representation of the miR-485-3p/NF-YB/Top2α axis in cells surviving the treatment of fludarabine. **B**, western blot analysis and quantification of NF-YB after fludarabine treatment. QRT-PCR analysis (**C**) and western blot analysis and quantification (**D**) of Top2α after fludarabine treatment. **E**, schematic representation of miR-485-3p/NF-YB-p53/MDR1 or CCNB2 axis in cells surviving treatment of fludarabine. **F**, representative immunoblot of p53 levels in cellular lysates of DU-145 cells treated (+) or untreated (-) with fludarabine (INPUT), in immunoprecipitates (IP) with anti NF-YB and anti-FLAG antibodies. **G**, relative expression level of CCNB2 and MDR1 in DU-145 cells treated with fludarabine versus untreated cells. All data were shown as S.D. from three independent experiments (*P<0.05, **P<0.01, ***P<0.001, unpaired *t*-test).

To investigate the mechanism(s) that mediate(s) the reduction of sensitivity to fludarabine, we looked at the molecular targets of miR-485-3p. Among them we focused on the β subunit of nuclear factor-Y (NF-YB), which is post-transcriptionally inhibited by miR-485-3p and is a transcriptional repressor of topoisomerase IIα (Top2α) [20] (Figure 4A). The immunoblot analysis showed that NF-YB was down regulated in fludarabine-treated cells (Figure 4B) and accordingly Top2α was up regulated at both the RNA (Figure 4C) and protein levels (Figure 4D).

Interestingly two other genes, cyclin B2 (CCNB2) [21] and MDR1 [22], have been reported to be repressed by NF-YB when associated with p53 (Figure 4E) [21]. Co-immunoprecipitation experiments demonstrated that NF-YB and p53 interact in DU-145 cells (Figure 4F). In accordance with this, we observed the down regulation of NF-YB and the up regulation of CCNB2/MDR1 (Figure 4G) in fludarabine-treated cells. A possible scenario is that cells harboring high levels of miR-485-3p were able to proliferate as the down regulation of the transcription factor NF-YB triggered the transcription of multiple pro-survival genes.

## Discussion

In this work we used DU-145 prostate cancer cells as an *in vitro* model to address whether the release of marker miRNAs by tumor cells is affected by treatment with a cytotoxic drug. Firstly, we showed that the prostate cancer cell line DU-145 spontaneously releases PCS-miRNAs in higher amounts than S-miRNAs in comparison to PNT1A cells. Secondly, we report that the released PCS-miRNAs were up regulated within cells, suggesting that in physiological conditions the release might represent a compensatory mechanism between the endogenous and the exogenous microenvironments. Thirdly, we show that fludarabine enhances the release into the growth medium of PCS-miRNAs but not of S-miRNAs, suggesting that the release under cytotoxic treatment was a regulated process. Finally, we report that the release/retention process is dependent on either the genetic background of the cells or the cytotoxic drug used.

Several studies have indicated that miRNAs can be actively secreted by living cells. Even if the secretory mechanism of miRNAs remains unclear so far, two different paths have been suggested: active secretion by a protein-miRNA complex or via cell-derived membrane vescicles (MVs) [23]. Among the MVs family we considered exosomes, small (40-100 nm) MVs of endocytic origin that contain a great variety of proteins, mRNAs and miRNAs [24,25]. A growing body of evidence indicates that exosomal miRNAs can be transferred from a donor to a recipient cell, mediating intercellular communication [26-28]. While exosomes are not a new discovery, recent evidence has indicated a wide range of application of exosomes research, including disease diagnosis [29-33] and prognosis [34], drug delivery [35] and more. We analyzed exosomes isolated from the growth medium of DU-145 cells either treated or untreated with fludarabine and found that the exosomes of treated cells (EXO/flu^+^) were more heavily loaded than the exosomes of untreated cells (EXO/flu^-^) for both PCS-miRNAs and S-miRNAs. These data seem to indicate that exosomes are used to export miRNAs from the intracellular to the extracellular environment and confirm that the release through exosomes is proportional to the intracellular expression level of each miRNA.

Intriguingly, miR-485-3p, in spite of its intracellular up regulation, was released less in the growth medium of fludarabine treated cells and, in addition, it was less loaded on EXO/flu+ than on EXO/flu^-^. The reduced loading was confirmed by transfecting DU-145 cells with either miR-485-3p or miR-100. We observed that both miRNAs were loaded on exosomes, however when transfected cells were treated with fludarabine, miR-485-3p but not miR-100 loading was impaired. As miR-485-3p was associated with exosomes as efficiently as miR-100 in untreated cells, whereas only miR-485-3p was less associated with exosomes in treated cells, the hypothesis was that fludarabine selected cells with a reduced ability to export miR-485-3p either free or associated with exosomes.

Indeed, we showed that fludarabine surviving cells harvested at several days after treatment were stably overexpressing miR-485-3p suggesting that this miRNA was involved in survival. To highlight a possible mechanisms through which the high expression levels of miR-485-3p favored the survival of DU-145 cells treated with fludarabine, we looked at the molecular targets of miR-485-3p. It has been reported that miR-485-3p post-transcriptionally regulates NF-YB, a direct transcriptional repressor of Top2α gene [20] and of MDR1 and CCNB2 genes when associated to p53 [21,22]. We observed that cells surviving the treatment of fludarabine showed enhanced expression of miR-485-3p, down regulation of NF-YB and the activation of Top2α, MDR1 and CCNB2 genes suggesting that cells were less sensitive to fludarabine as they had this pathways activated.

## Conclusions

In this paper we demonstrate that prostate cancer secretory miRNAs were released in the growth medium of prostate cancer cells either untreated or treated with fludarabine and that exosomes were involved in the export of miRNAs from the intra to the extracellular environment. We also report that miR-485-3p was highly expressed but poorly released in DU-145 cells surviving the cytotoxic treatment and that DU-145 cells harboring high expression level of miR-485-3p were able to survive the treatment with fludarabine probably because miR-485-3p mediated the activation of a network of prosurvival genes.

## Materials and methods

### Reagents

miRNeasy mini kit, QuantiTect Reverse Trascription Kit, QuantiTect SYBER Green PCR kit, miScript Reverse Transcription Kit, miScript SYBR Green PCR Kit, Polyfect Transfection Reagent (QIAGEN, Hilden, Germany); RPMI 1640 medium, Ham’s nutrient mixture F-12 (EuroClone); crystal violet, anti α-tubulin, anti FLAG M2, Hoechst 33258 (Sigma-Aldrich Corporation, Seelze, Germany); ECL, Hybond-C extra membranes (GE Healthcare, Cleveland, Ohio); anti Top2α (BD Biosciences, San Jose, California); anti NF-YB (GeneSpin, Milan, Italy); anti-CD63, HRP-conjugated secondary antibodies (Santa Cruz Biotechnology, Dallas, Texas); anti p53 (DakoCytomation, Glostrup, Denmark); TMRE, DiIC18 (Invitrogen, Life Technologies, Carlsbad, California); Dynabeads Protein G (Novex, Life Technologies, Carlsbad, California); fludarabine (Teva, Petah Tikva, Israel); taxotere (Taxotere, Sanofi Aventis, Milan, Italy); single stranded cel-miR-39, miR-485-3p, miR-100, miR-NC, d-485-3p, d-NC (GenePharma, Shanghai, China).

### Cells and culture conditions

DU-145, PNT1A, 22Rv1 and LNCaP cell lines were grown in RPMI 1640 medium. PC-3 were grown in Ham’s F-12. In all media 10% FBS, 1% penicillin/streptomycin (2 mM) and 1% L-glutammine (2 mM) were added to all media. Cells were incubated at 37°C in a humidified atmosphere containing 6% CO_2_. For fludarabine treatment, cells were grown for 24 h and treated with 10 μg/ml for 48 h (DU-145 and PC-3 cells) or 30 μg/ml for 48 h (22Rv1 and LNCaP). For taxotere treatment, cells were grown for 24 h and treated with 10nM taxotere for 48 h.

### Transfection of mature miRNAs and antagomiRNAs

Approximately 2×10^5^ cells were seeded in each well of a 6-well plate. After 24 h, cells were transfected with Polyfect Transfection Reagent according to the manufacturer instructions. We transfected the following oligonucleotides: either miR-485-3p (5′-GUCAUACACGGCUCUCCUCUCU-3′; 5′-AGAGAGGAGAGCCGUGUA UUUC-3′) or miR-100 (5′-AACCCGUAGAUCCGAACUUGU-3′; 5′-CAAGUUCGGAUCUACG GAUUAU-3′); antagomiRNA-485-3p (d-485-3p) (5′-AGAGAGGAGAGCCGUGUAUGACT-3′). As control we used either miR-NC (5′-UUCUCCGAACGUGUCACGUTT-3′; 5′-ACGUGAC ACGUUCGGAGAATT-3′) or antagomiRNA-NC (d-NC) (5 –CAGUACUUUUGUGUAGUAC AA-3′). After 6H hours from transfection, we added fresh growth medium and treated cells with fludarabine at 10 μg/ml.

### Extraction of RNA from the growth medium

Total RNA was extracted from 200 μl growth medium using the miRNEasy mini kit following the manufacturer recommendations (Purification of RNA from serum or plasma, Qiagen supplementary Protocol). According to the protocol, 5 μM cel-miR-39 (5′ -UCACCGGGUGUAAAUCAGCUUG- 3′) was added after the Qiazol step of the extraction protocol.

### Cell proliferation

Cell proliferation was measured as follows: 2×10^5^ cells per 6 well plate were seeded and after the treatment with fludarabine or treatment plus transfection, cells were fixed in 2% paraformaldehyde in PBS and subsequently stained with 0.1 % crystal violet dissolved in 20% methanol and let dry at room temperature. Cells were then lysed with 10% acetic acid and the optical density (OD 590 nm) of the solution, detected with Plate Reader apparatus (SpectraCount, Packard), was used to measure cell proliferation.

### Cell cycle

At specified time points cells were stained with propidium iodide and the cell cycle analyzed using a FACScalibur cytofluorimeter (BD Biosciences, San Jose, California). The percentage of cells in each phase was determined and the fold change of treated in comparison to untreated cells was calculated.

### Apoptosis assay

Apoptosis was detected using the cationic dye TMRE, a fluorescent probe that measures the loss of mitochondrial membrane potential associated with apoptosis [36]. Briefly, DU-145 treated or untreated cells were harvested, incubated with 100 nM TMRE for 40 min at 37°C in the dark and then analyzed by flow cytometry. The percentage of apoptotic cells was measured from the SSC density plots obtained.

### Plating efficiency

Cells exposed to fludarabine for 48 h were collected, counted and seeded at cell density of 200 cells/100 mm diameter culture dish to allow colony formation. After 10-12 days colonies were detected by staining dishes with 0.1 % crystal violet. Plating efficiency (no colonies/no seeded cells) of treated cells was normalized to that of the untreated cells.

### Quantification of miRNAs and mRNAs with Real-time PCR

Total RNA was extracted from 1×10^6^ cells using the miRNeasy mini kit following the manufacturer’s recommendations. To quantify multidrug resistance gene 1 (MDR1), Top2α and cyclin B2 (CCNB2), 1μg of total RNA was reverse transcribed using the QuantiTect Reverse Trascription Kit. Real-time PCR (qRT-PCR) was carried out with Rotor-Gene Q 2-Plex (Qiagen, Hilden, Germany) using the QuantiTect SYBR Green PCR kit. To quantify miRNAs, 1 μg of total RNA or 5-10 μl of RNA extracted from 200 μl growth medium were retrotranscribed with miScript Reverse Transcription Kit and qRT-PCR was carried out using the miScript SYBR Green PCR Kit. All reactions were performed in triplicate. Relative quantification of mRNAs and miRNAs expression was calculated with the fit point method. Transcript values were normalized to those obtained from the amplification of the internal control (glyceraldehyde 3-phosphate dehydrogenase (GAPDH) for mRNAs, U6 for intracellular miRNA and cel-miR-39 for extracellular miRNAs). The oligonucleotide sequences are reported in (Additional file 3: Figure S3).

### Western blot analysis

Equivalent amounts of proteins were resolved on SDS-PAGE gels at different acrylamide percentages and transferred to Hybond-C extra membranes by electroblotting. The resulting blots were blocked with 5% nonfat dry milk or BSA solution in TBST. Anti α-tubulin (1:20000), anti CD63 (1:500), anti Top2α (1:1000), anti NF-YB (1:1000) and anti p53 (1:5000) primary antibodies were used. Incubation was performed overnight at 4°C and bands were revealed after incubation with the recommended secondary antibody coupled to peroxidase using ECL. Scanned images were quantified using OptiQuant software and normalized to α-tubulin.

### Coimmunoprecipitation assay

1 mg of protein extracted from either DU-145 or DU-145 fludarabine treated cells were used in a coimmunoprecipitation assay. Sample were immunoprecipitated with 4 μg of either anti NF-YB or anti FLAG (as a control for aspecific immunoprecipitation) using Dynabeads Protein G following the manufacturers recommendations and separated on SDS-PAGE gels as described in the section Western Blot analysis.

### Isolation of exosomes

Exosomes were isolated according to Wang [37], with some modifications. Briefly, growth media were centrifuged at 2,000 × g for 30 min to remove cells and debris. The supernatant was transferred to a new tube and centrifuged at 10,000 × g for 45 min to further remove cells and debris. The supernatant was filtered through a 0.2 μm filter to remove particles larger than 200 nm (mainly microvesicles). Exosomes were then pelleted from microvesicles-depleted supernatant by centrifuging at 100,000 × g for 2 h. Exosomes were washed with PBS and resuspended in 50 μl PBS.

## Abbreviations

CCNB2: Cyclin B2; GAPDH: Glyceraldehyde 3-phosphate dehydrogenase; MDR1: Multidrug resistance gene 1; miRNA: microRNA; MV: Membrane vesicle; NF-YB: β subunit of nuclear factor-Y; PCS-miRNAs: Prostate cancer secretory miRNAs; qRT-PCR: Real-time PCR; S-miRNAs: Secretory miRNAs; Top2α: Topoisomerase IIα.

## Competing interests

The authors declare that they have no competing interests.

## Authors’ contributions

Participated in research design: SL, GR, MR. Conducted experiments: ME, SL, MR. Performed data analysis: MR. Wrote or Contributed to the writing of the manuscript: SL, GR, MR. All authors read and approved the final manuscript.

## Supplementary Material

Additional file 1: Figure S1 To investigate whether the retention/release of miR-485-3p was cell line dependent, we treated the androgen independent PC-3 (A), and two androgen dependent 22Rv1 (B) and LNCaP (C) prostate cancer cell lines with fludarabine. After 48 h treatment with a concentration of fludarabine able to inhibit cell proliferation and to leave surviving cells (PC3=10μg/ml; 22Rv1 and LNCaP=30μg/ml), growth media and cells were collected and the levels of intracellular and extracellular PCS- and S-miRNAs were evaluated. The dot plot of intracellular (Intra) and extracellular (Extra) miRNAs levels, expressed as fold changes in treated in comparison to untreated cells, showed that miR-485-3p was up regulated and released by PC3 (A) and 22Rv1 (B) and only release by LNCaP (C) cells suggesting that the retention/release was affected by the genetic background. We also tested whether the retention/release was strictly dependent on the univocal interaction between a given drug and genetic background. We treated DU-145 cells with 10nM taxotere, a specific drug for the treatment of prostate cancer, and after 48 h treatment growth medium and cells were collected and analysed as described above. We observed that the retention/release of miR-485-3p was unaffected (D) suggesting that the release of miR-485-3p was drug dependent. *Upper left quarter*: Intra down regulated, Extra up regulated; *Upper right quarter*: Intra up regulated, Extra up regulated; *Lower left quarter*: Intra down regulated, Extra down regulated; *Lower right quarter*: Intra up regulated, Extra down regulated. Click here for file

Additional file 2: Figure S2 *Upper panel*, western blot analysis of CD63 (a general exosomal marker) in exosomes isolated from treated or untreated DU-145 cells. The same volume of proteins extracted from exosomes isolated from treated and untreated cells were loaded. The Ponceau S staining was used as loading control*. Lower panel*, to be confident that the exosomes isolation procedure was able to isolate intact exosomes, we tested their functionality. Exosomes of DU-145 cells were labelled with DiIC18 according to Zhang [38], with some modifications. Briefly, DU-145 cells were labelled overnight with the lipophilic fluorescent tracer DiIC18. The culture medium was collected the following day and labelled exosomes were purified by the ultracentrifugation method (as described in *Materials and Methods* section). Purified exosomes were resuspended in RPMI 1640 medium supplemented with 10% FCS and added to the medium of exponentially growing DU-145 cells. Exosome uptake was monitored at 6, 12 and 24 h after their administration. At these time points cells were washed with PBS, fixed with PFA 2%, labelled with Hoechst stain and observed under fluorescence microscopy. A progressive increase of labelled cells was observed (from 6 h to 24 h). After 24 h almost all cells were labelled (right panel). White arrows indicate exosomes fused with the recipient cell membranes. Click here for file

Additional file 3: Figure S3 Oligonucleotides sequence. Click here for file
