# Supplementary figures and images for: Fludarabine treatment favors the retention of miR-485-3p by prostate cancer cells: implications for survival
Source: Mol Cancer. 2013 Jun 5;12:52. doi: 10.1186/1476-4598-12-52 (PMC3751825; doi:10.1186/1476-4598-12-52)

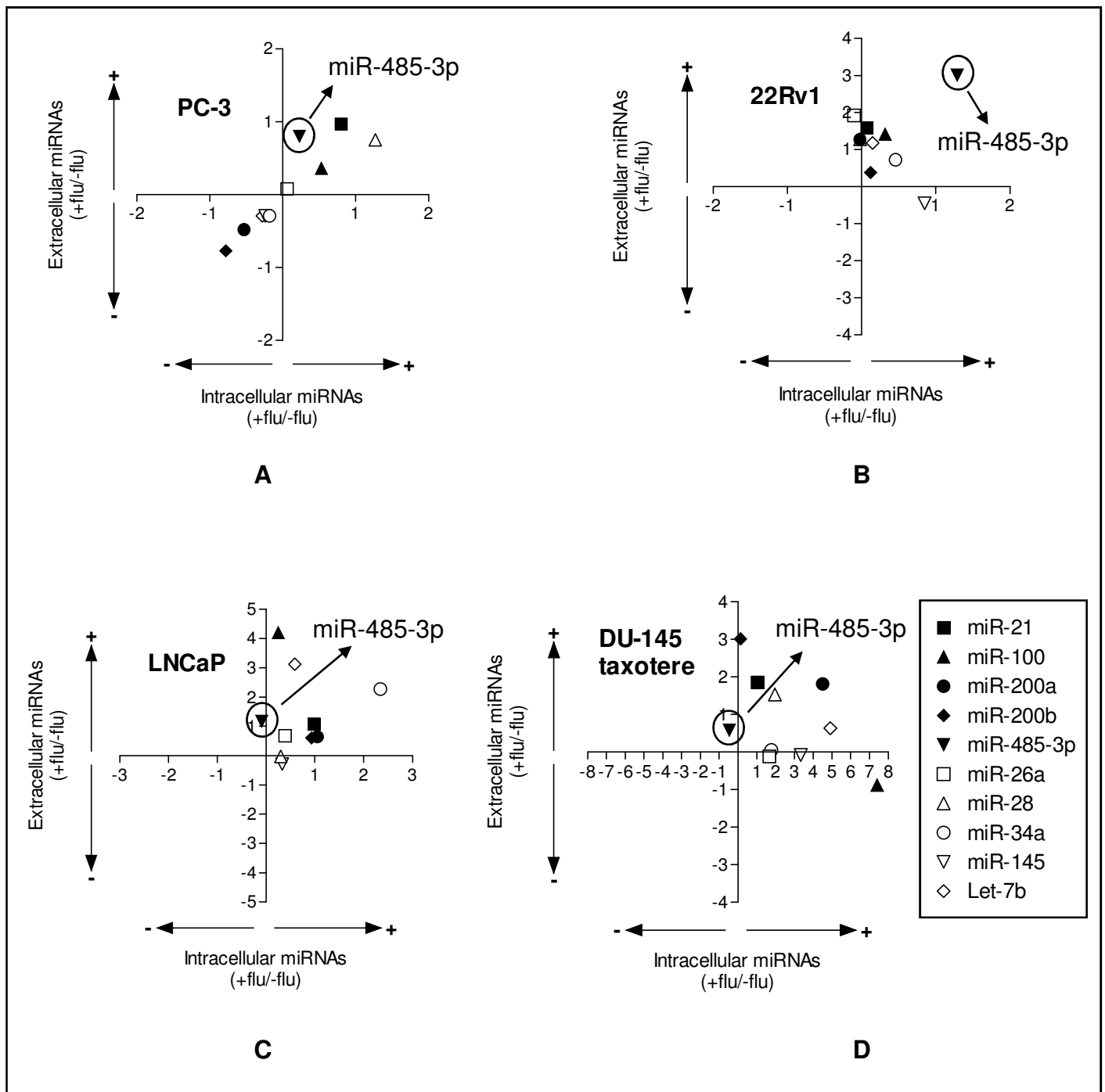

**Figure S1**

Supplement: Additional file 1: Figure S1 — To investigate whether the retention/release of miR-485-3p was cell line dependent, we treated the androgen independent PC-3 (A), and two androgen dependent 22Rv1 (B) and LNCaP (C) prostate cancer cell lines with fludarabine. After 48 h treatment with a concentration of fludarabine able to inhibit cell proliferation and to leave surviving cells (PC3=10μg/ml; 22Rv1 and LNCaP=30μg/ml), growth media and cells were collected and the levels of intracellular and extracellular PCS- and S-miRNAs were evaluated. The dot plot of intracellular (Intra) and extracellular (Extra) miRNAs levels, expressed as fold changes in treated in comparison to untreated cells, showed that miR-485-3p was up regulated and released by PC3 (A) and 22Rv1 (B) and only release by LNCaP (C) cells suggesting that the retention/release was affected by the genetic background. We also tested whether the retention/release was strictly dependent on the univocal interaction between a given drug and genetic background. We treated DU-145 cells with 10nM taxotere, a specific drug for the treatment of prostate cancer, and after 48 h treatment growth medium and cells were collected and analysed as described above. We observed that the retention/release of miR-485-3p was unaffected (D) suggesting that the release of miR-485-3p was drug dependent. Upper left quarter: Intra down regulated, Extra up regulated; Upper right quarter: Intra up regulated, Extra up regulated; Lower left quarter: Intra down regulated, Extra down regulated; Lower right quarter: Intra up regulated, Extra down regulated. [file 1476-4598-12-52-S1.pdf]

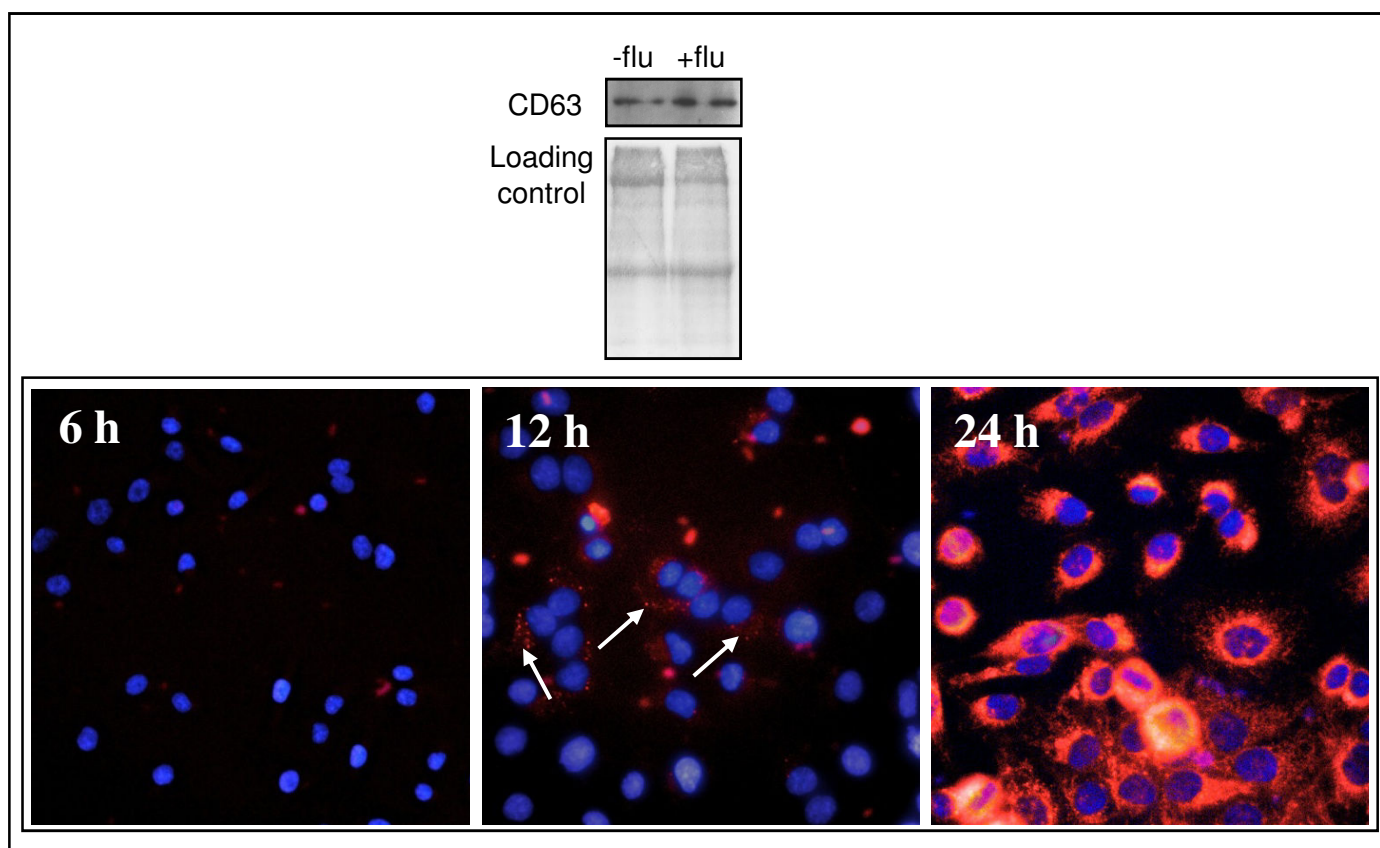

Figure S2

Supplement: Additional file 2: Figure S2 — Upper panel, western blot analysis of CD63 (a general exosomal marker) in exosomes isolated from treated or untreated DU-145 cells. The same volume of proteins extracted from exosomes isolated from treated and untreated cells were loaded. The Ponceau S staining was used as loading control. Lower panel, to be confident that the exosomes isolation procedure was able to isolate intact exosomes, we tested their functionality. Exosomes of DU-145 cells were labelled with DiIC18 according to Zhang [38], with some modifications. Briefly, DU-145 cells were labelled overnight with the lipophilic fluorescent tracer DiIC18. The culture medium was collected the following day and labelled exosomes were purified by the ultracentrifugation method (as described in Materials and Methods section). Purified exosomes were resuspended in RPMI 1640 medium supplemented with 10% FCS and added to the medium of exponentially growing DU-145 cells. Exosome uptake was monitored at 6, 12 and 24 h after their administration. At these time points cells were washed with PBS, fixed with PFA 2%, labelled with Hoechst stain and observed under fluorescence microscopy. A progressive increase of labelled cells was observed (from 6 h to 24 h). After 24 h almost all cells were labelled (right panel). White arrows indicate exosomes fused with the recipient cell membranes. [file 1476-4598-12-52-S2.pdf]
