# Supplementary material for: Fludarabine treatment favors the retention of miR-485-3p by prostate cancer cells: implications for survival
Source: Mol Cancer. 2013 Jun 5;12:52. doi: 10.1186/1476-4598-12-52 (PMC3751825; doi:10.1186/1476-4598-12-52)
Supplement: Additional file 3: Figure S3 — Oligonucleotides sequence. [file 1476-4598-12-52-S3.pdf]

| | Oligonucleotides sequence |
|---|---|
| miR-26a | 5'-TTCAAGTAATCCAGGATAGGCT -3‘ |
| miR-28 | 5'-AAGGAGCTCACAGTCTATTGAG-3’ |
| miR-34a | 5'-TGGCAGTGTCTTAGCTGGTTGT-3’ |
| miR-145 | 5'-GTCCAGTTTTCCCAGGAATCCCT-3’ |
| Let-7b | 5'-TGAGGTAGTAGGTTGTGTGGTT-3’ |
| miR-21 | 5'-TAGCTTATCAGACTGATGTTGA-3’ |
| miR-100 | 5'-AACCCGTAGATCCGAACTTGTG-3’ |
| miR-200a | 5'-CATCTTACCGGACAGTGCTGGA-3’ |
| miR-200b | 5'-CATCTTACTGGGCAGCATTGGA-3’ |
| miR-485-3p | 5'-GTCATACACGGCTCTCCTCTCT-3’ |
| cel-miR-39 | 5'-TCACCGGGTGTAAATCAGCTTG-3’ |
| U6 | 5'-CGCAAGGATGACACGCAAATTC-3' |
| NF-YB | Forward 5'-TCTCTGCAGACTATATTGGAGGAA-3‘<br>Reverse 5'-TCATGATCATTCATGCTGTCC-3' |
| Top2α | Forward 5'-CAACATGCCAATGAGTGAAA-3‘<br>Reverse 5'-GGACTTGGGCCTTAAACTTCA-3' |
| MDR1 | Forward 5'-AAGGCATTTACTTCAAACTTGTCA-3‘<br>Reverse 5'-TGGATTCATCAGCTGCATTTT-3' |
| CCNB2 | Forward 5'-TGGAAAAGTTGGCTCCAAAG-3‘<br>Reverse 5'-TCAGAAAAAGCTTGGCAGAGA-3' |
| GAPDH | Forward 5'-CACCAGCAGCGACTCTGA-3‘<br>Reverse 5'-GATCCAGACTCTGACCTTTTGC-3' |

**Figure S3**
